# Ectopic adrenocorticotropic hormone‐secreting carcinoid with solitary cryptococcosis in the lungs

**DOI:** 10.1002/cnr2.1731

**Published:** 2022-10-04

**Authors:** Hironori Takagi, Yuki Matsumura, Mitsuro Fukuhara, Sho Inomata, Hikaru Yamaguchi, Masayuki Watanabe, Yuki Ozaki, Satoshi Muto, Naoyuki Okabe, Yutaka Shio, Haruka Saito, Hayato Tanabe, Michio Shimabukuro, Hiroyuki Suzuki

**Affiliations:** ^1^ Department of Chest Surgery Fukushima Medical University Fukushima Japan; ^2^ Department of Chest Surgery Iwaki City Medical Center Iwaki Japan; ^3^ Department of Diabetes, Endocrinology and Metabolism Fukushima Medical University Fukushima Japan

**Keywords:** carcinoid, cryptococcosis, cryptococcus, ectopic adrenocorticotropic hormone‐secreting tumor, lung cancer

## Abstract

**Background:**

Carcinoid tumors can on rare occasions ectopically produce adrenocorticotropic hormone (ACTH), causing Cushing's syndrome, and patients could become immunocompromised. Care must therefore be taken regarding infectious complications. In particular, ACTH‐producing pulmonary carcinoid is not easy to diagnose by itself, and when combined with pulmonary nodules as infectious foci, each is very difficult to diagnose.

**Case:**

The patient was a 71‐year‐old woman with refractory diabetes. She showed clinical symptoms of Cushing's syndrome during treatment for diabetes and ectopic ACTH production was suspected based on biochemical and imaging tests. Nodules were identified in the left lung apex and lingual segment. Examination of resected nodules revealed that the nodule in the apex was pulmonary cryptococcosis, while the nodule in the lingual segment represented typical carcinoid. After surgery, clinical symptoms, laboratory findings, and diabetes all improved.

**Conclusion:**

We present this very instructive case in terms of the difficulty of diagnosing ACTH‐producing tumors, the possibility of infection complicating the immunodeficiency caused by ACTH‐producing tumors, and the surgical strategy.

## 1 INTRODUCTION

Ectopic adrenocorticotropic hormone (ACTH)‐producing tumors produce ACTH from sources other than the pituitary gland. Small cell lung carcinoma is the most common ectopic ACTH‐producing tumor, accounting for 45%–50%, followed by thymic carcinoid (15%–42%) and bronchopulmonary carcinoid (10%–40%).^1^, ^2^ Less than 1% of all pulmonary tumors are carcinoid tumors.^3^ Only 1%–5% of bronchopulmonary carcinoids produce ACTH.^4^ Another study specifically reporting on resected pulmonary carcinoids, 24.8% of tumors produced ACTH, and 17.4% of those tumors were associated with Cushing's syndrome.^5^ Carcinoid tumors are highly sensitive to detection by somatostatin receptor scintigraphy (SRS) because of the high expression of somatostatin receptors, but some cases may go undetected.^6^ In such cases, the utility of ^68^Ga‐DOTATATE imaging has been reported in recent years.^7^, ^8^ Cryptococcal infections usually occur in immunocompromised patients, and excessive secretion of ACTH and cortisol could theoretically cause immunodeficiency.^9^ In a previous report, disseminated cryptococcosis developed in a patient with pituitary Cushing's disease.^10^ We present a case of isolated pulmonary cryptococcosis due to immunodeficiency caused by an ectopic ACTH‐producing tumor and associated elevations in cortisol, in which preoperative diagnosis proved difficult and diagnosis from a surgical specimen was required. This case provides a valuable lesson in that when ACTH‐producing tumors are suspected and multiple nodules are present in the lungs, infectious lesions may be present along with carcinoid tumors.

### 1.1 Case

The patient was a 71‐year‐old woman. She did not keep any birds or animals, and had been treated for diabetes for over 30 years. She was on diet therapy and insulin therapy, but her diet appeared inadequate and her hemoglobin (Hb)A1c levels were within the range of 7.9%–8.5%. She was therefore admitted to her family hospital for dietary and lifestyle guidance in April 2019. During that hospitalization, she repeatedly developed pneumonia and bronchitis. In addition, findings of moon face, central obesity, and red skin lines led to a suspicion of Cushing's syndrome. During early morning fasting, plasma ACTH increased to 104 pg/mL (normal, 7.2–63.3 pg/mL) and serum cortisol increased to 23.5 μg/dL (normal, 6.2–19.4 μg/dL). In August 2019, at the time of consultation at our hospital (Fukushima Medical University Hospital), the patient showed: weight, 52.1 kg; height, 139.9 cm; body mass index, 26.6 kg/m^2^; blood pressure, 125/60 mmHg; and heart rate, 81 beats/min with regular rhythm. At this time, 24‐h urinary free cortisol (UFC) was slightly increased to 128 μg (normal, 20–100 μg).

We performed screening tests for Cushing's syndrome. No diurnal variation was seen in serum cortisol (values on 1 day: 8.15 μg/dL at 08:00; 12.30 μg/dL at 16:00; and 11.85 μg/dL at 23:00), and after a 0.5‐mg overnight dexamethasone suppression test, serum ACTH and cortisol were not suppressed (ACTH: 67.80 pg/mL; cortisol: 11.28 μg/dL). Based on these findings, ACTH‐dependent Cushing's syndrome was suspected and we performed testing to confirm the diagnosis. After 8‐mg overnight dexamethasone suppression testing, serum cortisol was suppressed (ACTH: 31.79 pg/mL; cortisol: 4.12 μg/dL). No response to ACTH was seen in the corticotropin‐releasing hormone (CRH) loading test (after CRH loading: basal plasma ACTH, 85.84 pg/mL; peak plasma ACTH, 87.18 pg/mL). Magnetic resonance imaging of the brain showed no abnormal enhancement of the pituitary gland.

**FIGURE 1:**
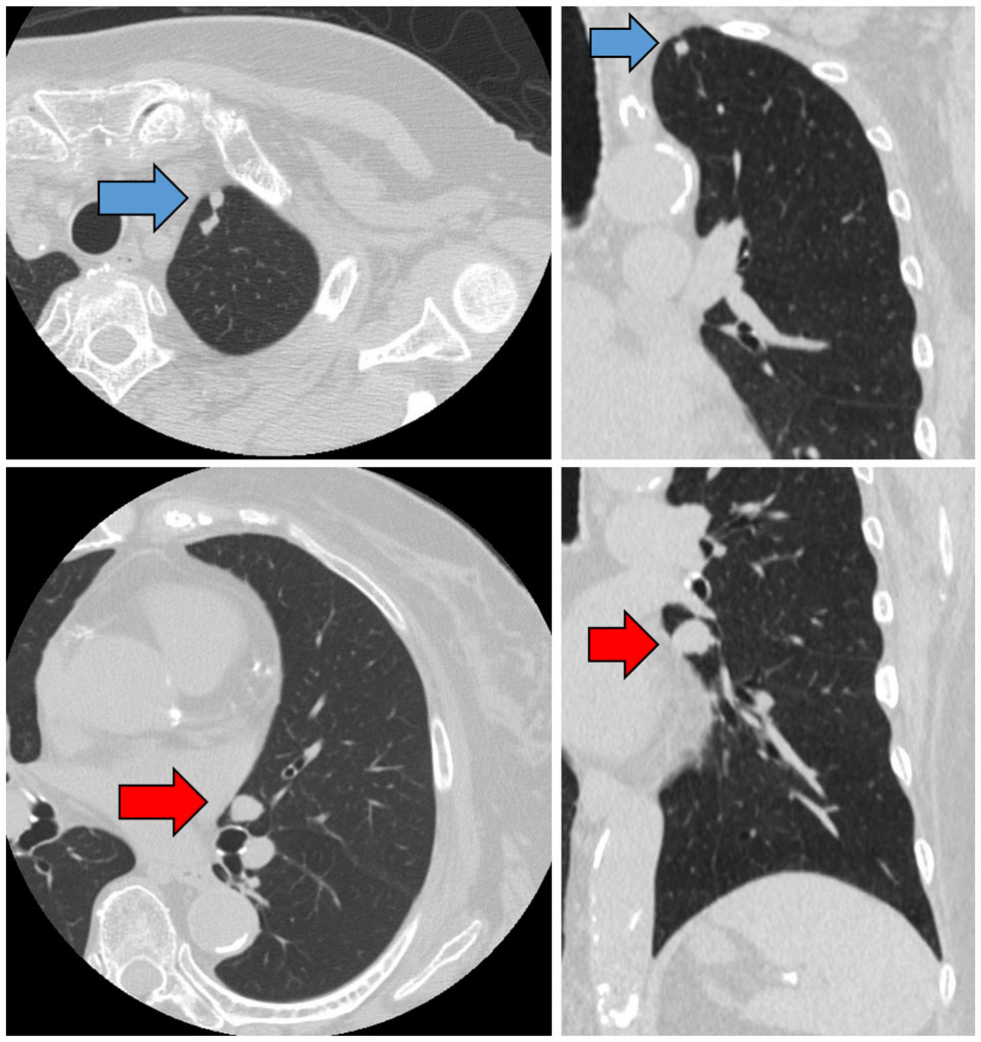
Computed tomography shows a 6‐mm nodule in the apex region (blue arrow) and a 15‐mm nodule in the lingual region (red arrows) of the left lung.

High‐resolution computed tomography (CT) showed two pulmonary nodules in the left lung apex and lingual regions (Figure 1). The results of blood tests were normal, showing: white blood cell count, 5.2 × 10^3^/μL; C‐reactive protein, 0.04 mg/dL; carcinoembryonic antigen, 4.9 ng/mL; squamous cell carcinoma antigen, 0.5 ng/mL; cytokeratin 19 fragment (CYFRA 21‐1), 3.09 ng/mL; and neuron‐specific enolase (NSE), 208 pg/mL. Only NSE was elevated. Positron emission tomography/CT showed no significant accumulations of ^18^F‐fluorodeoxyglucose in those lung nodules or other sites. SRS was performed using ^111^In‐octreotide, and no positive lesions were found throughout the entire body.

Although serum cortisol was suppressed in the high‐dose dexamethasone suppression test, Cushing's syndrome due to an ectopic ACTH‐producing tumor was suspected. However, the patient did not agree to selective venous sinus blood sampling from the cavernous sinuses or inferior petrosal sinus due to the invasiveness of the procedures. In addition, the possibility of ectopic ACTH‐producing tumor could not be ruled out due to the presence of lung tumors. However, CT findings suggested these tumors would be difficult to sample by bronchoscopic lung biopsy and we therefore performed surgical biopsy in December 2019.

With the patient under general anesthesia, two nodules in the left lung underwent wedge resection with free margins under thoracoscopy. Intraoperatively, the easily resectable pulmonary apex lesion underwent wedge resection first, and frozen section diagnosis revealed pyogenic granuloma. We therefore determined that the entire left lung was infected, and surgery was finished after adding only wedge resection of the lingual region.

**FIGURE 2:**
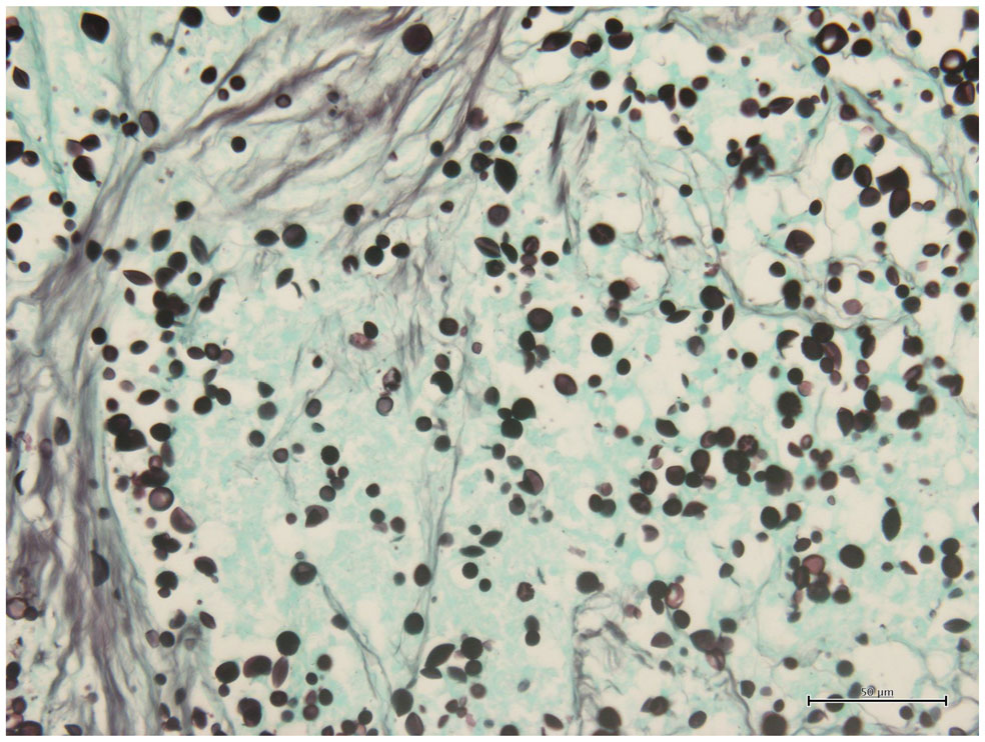
Tumor of the pulmonary apex. Many oval fungi are seen, and the diagnosis is pulmonary cryptococcosis (Grocott stain). Magnification, ×400.

**FIGURE 3:**
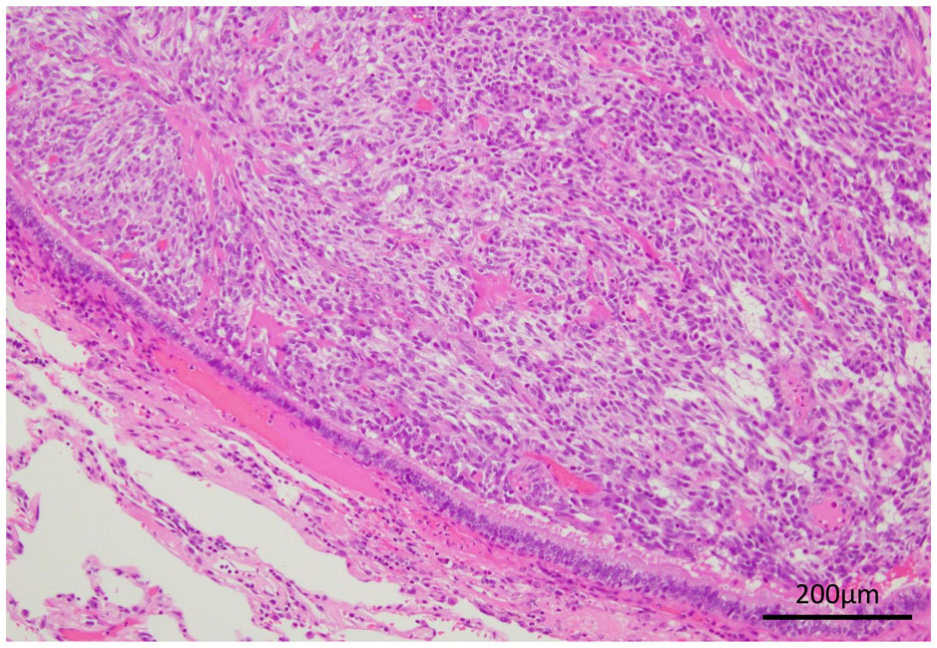
Tumor in the lingular area: Spindle cells proliferated in ribbon‐ and spore‐like patterns with hematoxylin and eosin stain. Magnification, ×200.

**FIGURE 4:**
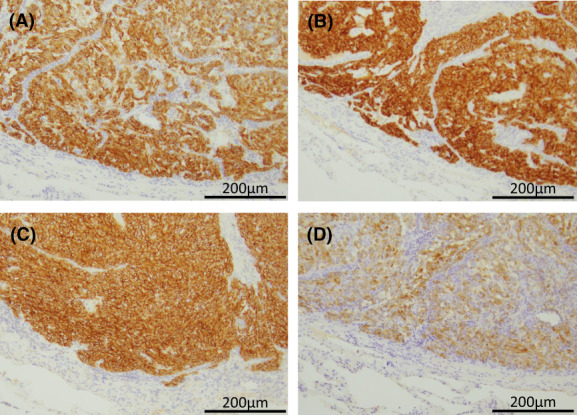
Immunohistochemistry reveals typical carcinoid with strong positivity for neuroendocrine markers (A: chromogranin A; B: synaptophysin; C: CD56) and ACTH (D). Magnification, ×200.

In the definitive diagnosis on histopathological examination of the resected specimens, the lesion in the left lung apex showed caseous necrosis, and no mycobacteria were observed on Ziehl–Neelsen staining. Grocott staining and periodic acid‐Schiff staining showed numerous oval‐shaped fungi (Figure 2) positive for mucicarmine staining, indicating fungal infection by *Cryptococcus*. Lesions in the lingular segment comprised spindle cells proliferating in ribbons and foci (Figure 3), and immunohistochemistry showed strong positivity for chromogranin A, synaptophysin, CD56, and ACTH (Figure 4). No necrosis was present in the tumor, and the mitosis count was 1/2 mm^2^. The patient was diagnosed with ACTH‐producing pulmonary typical carcinoid (p‐T1bN0M stage IA2) with pulmonary cryptococcosis. Although this was before the diagnosis of ACTH‐producing tumor, hydrocortisone was started at 50 mg/day immediately after surgery as a precaution. On postoperative day 4, basal plasma ACTH decreased to 6.67 pg/mL and serum cortisol levels decreased to 3.64 μg/dL. Although hydrocortisone might have had some effect, ACTH and cortisol remained at normal levels. Hydrocortisone dosage was gradually decreased to 20 mg/day, and the patient was discharged 15 days postoperatively. Subsequently, 24‐h UFC levels were also normal, diabetes control was improved, and clinical symptoms resolved. Finally, at 6 months postoperatively and on hydrocortisone at 15 mg/day, the 0.5‐mg dexamethasone suppression test suppressed ACTH and cortisol levels (ACTH: 14.55 pg/mL; cortisol: 0.288 μg/dL), confirming improvement of Cushing's syndrome. Furthermore, basal plasma concentration of ACTH was 20.05 pg/mL and cortisol was 10.32 μg/dL, so secretions were considered to be adequately maintained and hydrocortisone dose was further tapered and terminated 2 years postoperatively. Preoperatively, we did not test for cryptococcal antigen in the serum because we did not strongly suspect infection. Results for cryptococcal antigen in the serum examined 2 days postoperatively were negative. Cerebrospinal fluid was also examined after discharge, but no cryptococcus was found. The patient did not request additional lobectomy. As of July 2022, 18 months postoperatively, the patient was alive with no tumor recurrence and no clinical or laboratory evidence of elevated ACTH or Cushing's syndrome. She remains on insulin therapy for diabetes, but HbA1c is hovering around 7%. She patient is living independently and we will continue careful observation with laboratory data and CT.

## 2 DISCUSSION

In this case, the patient showed elevated levels of ACTH and cortisol and poorly controlled diabetes mellitus that was affected by ectopic ACTH‐producing typical carcinoid in the lingual region of the left lung. This was thought to have caused the patient to become immunocompromised and to have developed isolated pulmonary cryptococcosis in the left lung apex. Past reports have described cases of systemic infection due to the immunocompromised state caused by ectopic ACTH‐producing tumors,^10^ but cases of infection with isolated nodule only in the lungs, as in this case, are rare.^11^, ^12^ No elevated inflammatory response was seen preoperatively and sputum culture yielded negative results. On CT, nodules in the apex and lingual region appeared difficult to diagnose by bronchoscopic lung biopsy. Based on the elevated ACTH and cortisol levels and CT results, either or all of the nodules were suspected to be ACTH‐producing tumors. In recent years, the usefulness of ^68^Ga‐DOTATATE imaging as a test for diagnosing and localizing neuroendocrine tumors, including carcinoid, has been reported.^7^, ^8^ However, since our hospital lacks the equipment for ^68^Ga‐DOTATATE and the test is not covered by insurance in Japan, we performed the ^111^In‐octreotide scan. Unfortunately, the octreotide scan did not yield useful findings, but ACTH‐producing carcinoid tumor was still suspected, and surgery was performed to allow diagnosis from the resected specimens. Where available, ^68^Ga‐DOTATATE scans could be useful in determining treatment strategies, particularly in cases of suspected carcinoid tumors, including multiple pulmonary nodules, such as in the present case.

Intraoperative pathological evaluation of the tumor at the lung apex led to the suspicion of mycobacterial infection. The operation was therefore terminated with partial resection of two nodules from the lung. Since lobectomy would have been performed if pulmonary carcinoid had been diagnosed, the patient was carefully followed‐up postoperatively with endocrine and imaging tests. If a nodule is suspected to represent pulmonary carcinoid with ectopic ACTH production, a nodule of pulmonary infection may be present at the same time due to potential immunodeficiency. In cases such as this one, accurate preoperative diagnosis of each of the multiple nodules is difficult, so the surgical technique should be determined with the possibility that each nodule may show a different diagnosis, such as tumor or infection. Fortunately, no serious perioperative infections occurred in this case, but ACTH‐producing tumors can lead to an increased risk of infection, perioperative pneumonia and wound infection due to the immunocompromised state, so care must be taken. This case was very instructive in these respects.

## 3 CONCLUSION

When ectopic ACTH‐producing tumors are suspected and multiple nodules are present in the lungs, diagnosis may be difficult because of possible concomitant infectious lesions due to an easily infected condition and carcinoid tumor. In addition, the clinician should be aware of perioperative infections due to an immunocompromised state.

## AUTHOR CONTRIBUTIONS

**Yuki Matsumura:** Conceptualization (equal); data curation (equal); formal analysis (lead); investigation (equal); methodology (equal); project administration (supporting); supervision (lead); validation (lead); visualization (lead); writing – original draft (lead); writing – review and editing (lead). **MItsuro Fukuhara:** Formal analysis (supporting); resources (supporting); writing – review and editing (supporting). **Sho Inomata:** Formal analysis (supporting); resources (supporting); writing – review and editing (supporting). **Hikaru Yamaguchi:** Formal analysis (supporting); resources (supporting); writing – review and editing (supporting). **Masayuki Watanabe:** Formal analysis (supporting); resources (supporting); writing – review and editing (supporting). **Yuki Ozaki:** Formal analysis (supporting); resources (supporting); writing – review and editing (supporting). **Satoshi Muto:** Formal analysis (supporting); resources (supporting); writing – review and editing (supporting). **Naoyuki Okabe:** Formal analysis (supporting); resources (supporting); writing – review and editing (supporting). **Yutaka Shio:** Formal analysis (lead); resources (supporting); writing – review and editing (supporting). **Haruka Saito:** Formal analysis (supporting); resources (supporting); writing – review and editing (supporting). **Hayato Tanabe:** Conceptualization (supporting); formal analysis (lead); resources (lead); supervision (lead); writing – original draft (supporting); writing – review and editing (lead). **Michio Shimabukuro:** Conceptualization (equal); formal analysis (equal); investigation (equal); resources (equal); supervision (lead); writing – original draft (supporting); writing – review and editing (equal). **Hiroyuki Suzuki:** Conceptualization (lead); formal analysis (lead); investigation (lead); project administration (lead); supervision (lead); writing – original draft (supporting); writing – review and editing (lead).

## CONFLICT OF INTEREST

This report did not receive any other specific grant from funding agencies in the public, commercial, or not‐for‐profit sectors. None of the authors have any commercial or financial involvements in connection with this study that represent or appear to represent any conflicts of interest.

## ETHICAL STATEMENT

Institutional approval was not required for this case report. All patient information was deidentified for the purposes of this case report. Informed consent for publication was obtained from the patient.

## Data Availability

Data sharing is not applicable to this article as no new data were created or analyzed in this study.
